# Identification of key regulatory genes involved in the sporophyte and gametophyte development in *Ginkgo biloba* ovules revealed by in situ expression analyses

**DOI:** 10.1002/ajb2.1862

**Published:** 2022-05-19

**Authors:** Greta D'Apice, Silvia Moschin, Sebastiano Nigris, Riccardo Ciarle, Antonella Muto, Leonardo Bruno, Barbara Baldan

**Affiliations:** ^1^ Botanical Garden University of Padova Padova 35123 Italy; ^2^ Department of Biology University of Padova Padova 35131 Italy; ^3^ Department of Biology Ecology and Earth Sciences (DiBEST), University of Calabria, Arcavacata of Rende CS 87036 Italy

**Keywords:** gametophyte development, *Ginkgo biloba*, *Ginkgo biloba* ovule, in situ hybridization, ovule development, ovule integument, ovule regulatory genes, seed development, sporophyte development

## Abstract

**Premise:**

In *Arabidopsis thaliana*, the role of the most important key genes that regulate ovule development is widely known. In nonmodel species, and especially in gymnosperms, the ovule developmental processes are still quite obscure. In this study, we describe the putative roles of *Ginkgo biloba* orthologs of regulatory genes during ovule development. Specifically, we studied *AGAMOUS* (*AG*), *AGAMOUS‐like 6* (*AGL6*), *AINTEGUMENTA* (*ANT*), *BELL1* (*BEL1*), *Class III HD‐Zip*, and *YABBY Ginkgo* genes.

**Methods:**

We analyzed their expression domains through in situ hybridizations on two stages of ovule development: the very early stage that corresponds to the ovule primordium, still within wintering buds, and the late stage at pollination time.

**Results:**

*GBM5* (*Ginkgo* ortholog of *AG*), *GbMADS8* (ortholog of *AGL6*) and *GbC3HDZ1‐2‐3* were expressed in both the stages of ovule development, while *GbMADS1*, *GbAGL6*‐like genes (orthologs of *AGL*6), *GbBEL1‐2* and *YABBY Ginkgo* orthologs (*GbiYAB1B* and *GbiYABC*) seem mostly involved at pollination time. *GbANTL1* was not expressed in the studied stages and was different from *GbANTL2* and *GbBEL1*, which seem to be involved at both stages of ovule development. In *Ginkgo*, the investigated genes display patterns of expression only partially comparable to those of other studied seed plants.

**Conclusions:**

The expression of most of these regulatory genes in the female gametophyte region at pollination time leads to suggest a communication between the sporophytic maternal tissue and the developing female gametophyte, as demonstrated for well‐studied model angiosperms.

The main molecular mechanisms and genes responsible for ovule development have been largely studied in *Arabidopsis thaliana*, in which the mature ovule is made by the female gametophyte surrounded by two integuments, as in the majority of angiosperms. *Arabidopsis* ovule primordia arise from placental tissue, and their subsequent development are due to the cooperation of several genes. Among them, the MADS‐box genes encoding for the transcription factors SEEDSTICK (STK), AGAMOUS (AG), and SHATTERPROOF 1 and 2 (SHP1‐2) redundantly promote ovule identity (Pinyopich et al., 2003). AINTEGUMENTA (ANT), which encodes for an AP2‐like transcription factor, is specifically required for the development of the integuments and the female gametophyte (Elliot et al., 1996; Krizek 1999; Mizukami and Fischer, 2000). Subsequently, *ANT* expression in the integument initiation region activates the emergence of ovule integuments from the chalazal pole of ovule primordia (Cucinotta et al., 2020). BELL1 (BEL1) then activates the expression of *INNER NO OUTER* (*INO*), an angiosperm specific gene belonging to the *YABBY* family, which is required for the asymmetric growth of the ovule outer integument (Reiser et al., 1995; Villanueva et al., 1999). After the determination and the initiation of the primordia of the ovule integuments, they grow to cover the nucellus. This process is mainly regulated by SHORT INTEGUMENTS1 (SIN1), which promotes proper cell elongation (Robinson‐Beers et al., 1992; Schauer et al., 2002; Barro‐Trastoy et al., 2020). Class III HD‐Zip and KANADI transcription factors are required for the proper determination of the adaxial‐abaxial polarity of the two growing integuments (Eshed et al., 2001; McAbee et al., 2006; Kelley et al., 2009; Kelley and Gasser, 2009). Moreover, ABERRANT TESTA SHAPE (ATS/KAN4) is responsible for the development of the inner integument and the separation layer between the two integuments (McAbee et al., 2006; Gasser and Skinner, 2019). Although the studies conducted on *Arabidopsis* and on other angiosperms describe extensively the molecular patterns controlling ovule development, deeper studies in gymnosperms are still largely missing.

*Ginkgo biloba* has been chosen because of its isolated phylogenetic position and the availability of its genome. Recently, it has been demonstrated that the processes that drive the seed coat development in *Ginkgo* are activated upon pollination, with the activation of lignin and fatty acids biosynthesis pathways, which is necessary for the differentiation of the seed coat layers (D'Apice et al., 2021). Nevertheless, the early mechanisms and genes that drive and regulate the early ovule development are still poorly studied. It has already been demonstrated that the MADS‐box genes *GBM5*, *GbMADS1*, *GbMADS8*, and *GbMADS11* (respectively, orthologous of *AGAMOUS*, *AGL6*, *AGL6*, and *TM8*‐like genes) are expressed in *Ginkgo* ovules (Lovisetto et al., 2012). Moreover, from the study conducted by Wang et al. (2016) it emerged that orthologs of genes commonly associated with ovule development in angiosperms (such as *WUSCHEL*, *EARLY FLOWERING 3*, *AINTEGUMENTA*, *BELL1*) are detectable in developing *Ginkgo* ovules, revealing that gymnosperms and angiosperms might share similar gene regulatory pathways during the ovule development (Wang et al., 2016).

In this study, we performed in situ hybridizations of *AG*, *AGL6*, *ANT*, *BEL1*, *Class III HD‐Zip*, and *YABBY* genes in *Ginkgo* buds containing ovule and leaf primordia, and in just‐pollinated ovules. Some of these regulatory genes have already been investigated in *Ginkgo*, mostly regarding diverse organs (not only ovules) and stages of development (Floyd et al., 2006; Lovisetto et al., 2012; Finet et al., 2016; Zumajo‐Cardona et al., 2021). Here, we describe the expression pattern of these genes in an early stage of ovule development and during the crucial stage of pollination (respectively corresponding to stages 4 and 8 as described in D'Apice et al., 2021), highlighting the changes in their expression domains. This study provides an overview of the gene network controlling *Ginkgo* early ovule development, expanding the knowledge about ovule developmental programs in nonmodel seed plants.

## MATERIALS AND METHODS

### Investigated genes

*Ginkgo* genes that have been studied in this work are reported in Table 1. Some of them have been already identified as putative orthologs of regulatory genes involved in the process of ovule development. Information about their annotation, sequence identification (ID), and previous studies characterizing them are given in Table 1. *GbBEL1‐2* was identified through a Basic Local Alignment Search Tool (BLAST) search in *Ginkgo* CDS (available from Guan et al., 2016) using as query the *Arabidopsis BEL1* gene (accession number NM_123506.3). *GbAGL6*‐like was first identified as belonging to the *AGL6* clade by Wan et al. (2016) and since grouped with the *Pinus tabuliformis PtDAL14* (Carlsbecker et al., 2004). Primer sequences used to amplify target regions of these genes to perform expression analyses (reported below) are listed in Appendix S1.

**Table 1:** *Ginkgo biloba* genes studied in this work. Information about gene type, coding sequence (CDS) reference code to the genome (2016) and GenBank sequence ID if previously characterized are reported. n.a., not available.

Gene type	CDS code in *Ginkgo* genome (Guan et al., 2016)	GenBank sequence ID	Previous identifications or studies	Gene name in this work
*AINTEGUMENTA*	Gb_05487	AB195245.1	***GbANTL1*** (Shigyo et al., 2006)	*GbANTL1*
*AINTEGUMENTA*	Gb_07049	n.a.	***GibiANT*** (Zumajo‐Cardona et al., 2021)	*GbANTL2*
*BELL1*	Gb_36166	n.a.	***GibiBEL1*** (Zumajo‐Cardona et al., 2021)	*GbBEL1*
*BELL1*	Gb_39741	n.a.	n.a.	*GbBEL1‐2*
*AGAMOUS*	Gb_16301	AY114304.1	***GBM5*** (Jager et al., 2003)	*GBM5*
*AGL6*	Gb_41549	KX061105.1	***MADS‐box transcription factor 6*** (Wan et al., 2016)	*GbAGL6*‐like
*AGL6*	Gb_36364	AB029463.1	***GbMADS1*** (Lovisetto et al., 2012)	*GbMADS1*
*AGL6*	Gb_28337	AB029470.1	***GbMADS8*** (Lovisetto et al., 2012)	*GbMADS8*
*Class III HD‐Zip*	Gb_18245	DQ385525.1	***GbC3HDZ1*** (Floyd et al., 2006)	*GbC3HDZ1*
*Class III HD‐Zip*	Gb_22761	DQ385526.1	***GbC3HDZ2*** (Floyd et al., 2006)	*GbC3HDZ2*
*Class III HD‐Zip*	Gb_02083	DQ385527.1	***GbC3HDZ3*** (Floyd et al., 2006)	*GbC3HDZ3*
*YABBY*	Gb_22423	LN871573.1	***GbiYAB1B*** (Finet et al., 2016)	*GbiYAB1B*
*YABBY*	Gb_08229	LN871575.1	***GbiYABC*** (Finet et al., 2016)	*GbiYABC*

### Phylogenetic analyses

Because only a few phylogenetic analyses were present in the literature, or the available analyses did not include *Ginkgo* sequences in their data set, we performed phylogenetic analyses of YABBY and ANT protein sequences.

To construct the data set, a similarity‐based search was conducted through BLAST (https://blast.ncbi.nlm.nih.gov/blast.cgi) using *Ginkgo* nucleotide and protein sequences (genome source deposited by Guan et al., 2016) as queries. This search had the primary objective of finding similar gymnosperm sequences. To enrich this gymnosperm data set with a heterogeneous group of angiosperm sequences, the sequences of the target genes of model species were retrieved from the U.S. National Center for Biotechnology Information (NCBI) and European Molecular Biology Laboratory–European Bioinformatics Institute (EMBL‐EBI, https://www.ebi.ac.uk) databases and then used as queries in a BLAST similarity search as well. Some sequences were also retrieved from the 1 KP database (https://db.cngb.org/onekp). The data sets used for the two phylogenetic analyses are reported in Appendices S2 and S3. Sequence alignments were performed with the online Multiple Alignment using Fast Fourier Transform (MAFFT) tool (https://mafft.cbrc.jp/alignment/server) (Katoh et al., 2019). The alignment in output was visualized through Jalview (Clamp et al., 2004) and further refinements were made if necessary. When performing explorative nucleotide sequence alignments, jModelTest2 (Posada, 2008; Darriba et al., 2012) was used to determine the best‐fit model of nucleotide substitution. When the alignment was made up of amino‐acid sequences ModelTest‐NG (Darriba et al., 2020) was used instead. Refined alignments and the best model found were then used for phylogenetic tree reconstructions, run on the Cyberinfrastructure for Phylogenetic Research (CIPRES) portal online (https://phylo.org). We used the RAxML‐HPC2 tool running on XSEDE with 1000 replicas of bootstrap.

### Plant material

*Ginkgo biloba* samples were collected from centuries‐old trees at the Botanical Garden of Padova, Italy. We sampled ovules at stages 4 and 8 according to the morphological description of ovule development provided in D'Apice et al. (2021). At stage 4, ovule primordia are contained within buds together with leaf primordia. The ovule integument is growing to enclose the already discernible nucellus. At stage 8, ovules measure about 2 mm in diameter and are ready to be pollinated. Samples were collected in parallel for total RNA extraction and in situ hybridization experiments. Samples for RNA extraction were immediately frozen in liquid nitrogen and stored at –80°C, while samples for in situ hybridization experiments were processed as follows in the next section.

### Sample fixation, embedding, and sectioning

Fresh samples were fixed in a 4% paraformaldehyde solution in 1X phosphate buffered saline (PBS) with mild vacuum infiltration, and maintained in fixative overnight at 4°C. Then, Samples were washed twice with 1X PBS (for 30 min each wash), and dehydrated using an ethanol series (30%, 50%, 70%, 85%) with 1 h for each step, followed by 95% ethanol overnight, and finally, two 100% ethanol stages for 30 min each. After the last dehydration step, ethanol 100% was gradually replaced with xylene series (1:3; 1:1; 3:1; 4:0; 4:0 xylene:ethanol, for 1 h each). Xylene was gradually replaced by Paraplast Plus (Leica Biosystems, Milan, Italy) (as described by Douglas et al., 2007). *Ginkgo* samples were embedded within steel base molds and maintained in plastic embedding rings at 4°C until they were processed. Sections of 8–10 µm were cut on the microtome Leica RM 2125 RTS (Leica Biosystems, Milan, Italy).

### RNA extraction, purification, and quantification

Total RNA was extracted using the protocol described by Chang et al. (1993), quantified using a NanoPhotometer (Implen GmbH, Munich, Germany), and treated with DNase I (New England Biolabs; Ipswich, Massachusetts, USA) to remove contaminating DNA. DNase I was removed through the RNA Clean & Concentration‐5 kit (Zymo Research, California, USA). RNA was quantified again and then conserved at –80°C until use.

### Probe synthesis and in situ hybridization protocol

One microgram of total RNA per sample was retro‐transcribed using the Invitrogen SuperScript III kit (Invitrogen, Waltham, Massachusetts, USA).

Gene‐specific probes for hybridizations were designed on the target gene sequences (Table 1) and obtained through RNA synthesis using a polymerase chain reaction (PCR)‐derived DNA templates. DNA templates were amplified from cDNA samples by Wonder Taq Polymerase (EuroClone, Milan, Italy), using primers listed in Appendix S1. PCR products were purified with the PureLink PCR Purification Kit (Invitrogen, Waltham, Massachusetts, USA). RNA Digoxigenin (DIG)‐labeled antisense and sense probes were synthetized from purified PCR amplicons by using T7 RNA polymerases (Roche, Rotkreuz, Switzerland) according to the manufacturer's protocol. The reaction mix contained the DIG RNA labeling mix (Roche, Rotkreuz, Switzerland) and the RNase inhibitor RNaseOUT (Invitrogen, Waltham, Massachusetts, USA).

We used the hybridization procedure described by Ambrose et al. (2000). The probes hybridizations were performed at 55°C overnight in 50% formamide‐humidified box. The antibody Anti‐Digoxigenin‐AP Fab fragments (Roche, Rotkreuz, Switzerland) was diluted 1:700 and incubated for 90 min at room temperature, and the detection with the two NBT/BCIP chromogens (Promega, Madison, Wisconsin, USA) was performed overnight. After staining was stopped, the slides were dehydrated, dried, and permanently mounted using Entellan New (Merck, Darmstadt, Germany). Slides were observed and photographed with a Leica DM500 optical microscope (Leica Biosystems, Milan, Italy).

## RESULTS

### Phylogenetic analyses

In Appendix S4, the YABBY tree highlights several well‐supported clades. The group containing mostly YABBY2 sequences is sister to the CRABS CLAW (CRC) group, both of which do not contain any gymnosperm sequence. INNER NO OUTER (INO) is another group not present in gymnosperms (highlighted in green in Appendix S4). However, gymnosperms, represented mostly by conifers plus *Ginkgo*, form a group that is close to the INO clade. Indeed, this group of gymnosperm sequences (highlighted in light blue in Appendix S4) is sister to the group made of INO clade plus two YAB sequences of cycads. Of great interest to us, the *Ginkgo* GbiYAB1B (Gb_22423) falls within this group and appears to be the closest putative *Ginkgo* ortholog to the *INO* gene. Gb_CTQ352481 is a shorter segment of the same sequence retrieved from the NCBI database. The other two YABBY sequences of *Ginkgo* are GbiYABA (Gb_36880) and GbiYABC (Gb_08229). They fall into a clade of gymnosperm sequences that is sister to the clade containing both the INO and the GbiYAB1B groups (Appendix S4). YABBY5 is another well‐supported group of the tree (highlighted in yellow in Appendix S4), and like YABBY2, it is not present in gymnosperm sequences.

In the AINTEGUMENTA tree (Appendix S5), gymnosperm sequences group separately from the angiosperm sequences (except for the *Amborella trichopoda* sequence XP_011625364.2) and each of the two ANT *Ginkgo* sequences is sister to a cluster of gymnosperm sequences, arguing that it is likely that there are two *AINTEGUMENTA* orthologs in gymnosperms. These two orthologs seem sufficiently different at the sequence level to be well divided into two distinct clades (Appendix S5).

### Expression patterns of regulatory genes in *Ginkgo* ovules at two stages of development

**Figure 1:**
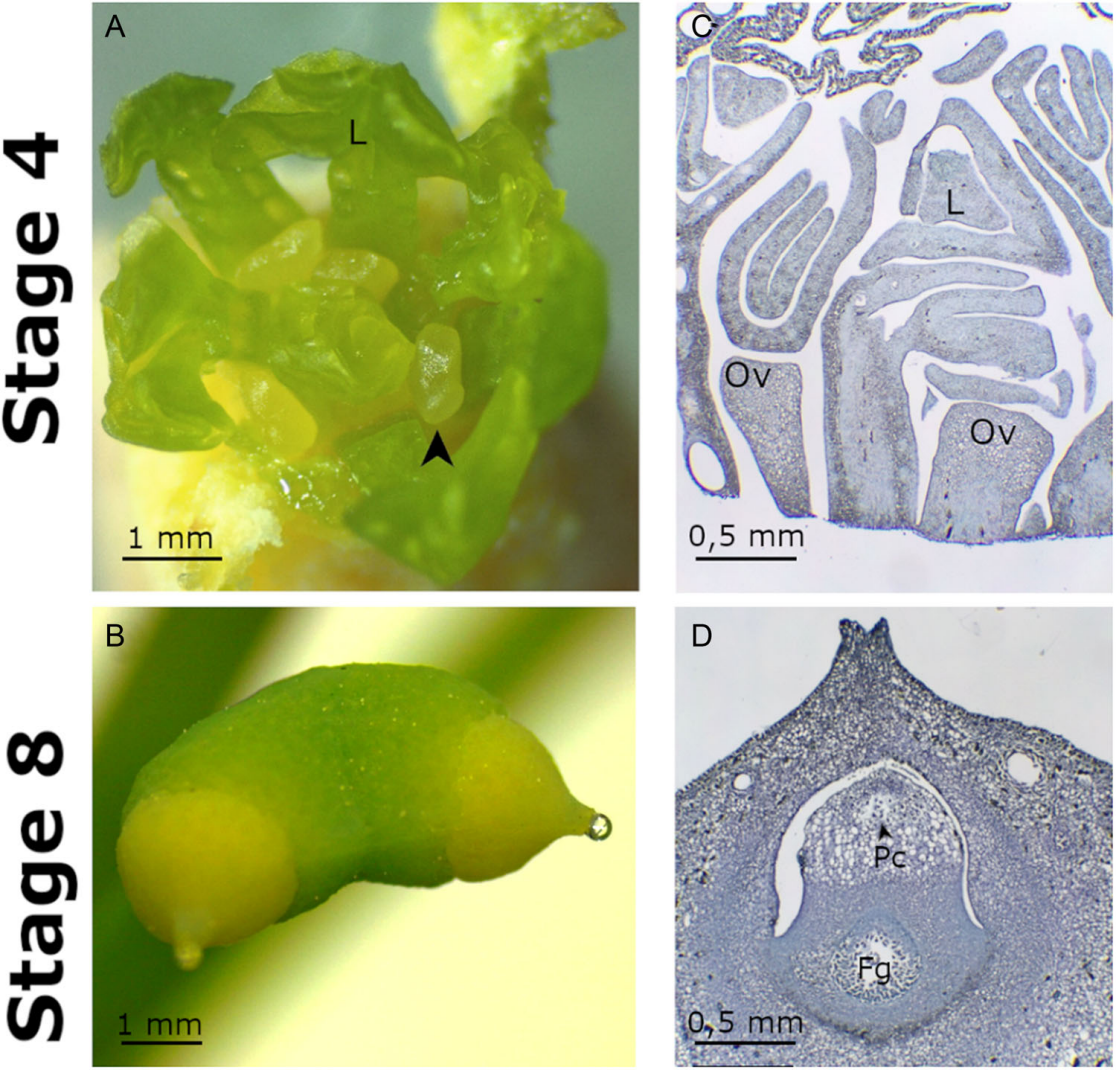
Stages 4 and 8 of *Ginkgo biloba* ovule development. (A) Fresh dissected bud in which most external bracts have been removed. Black arrowhead indicates one of the ovule primordia. (B) Fresh ovules at stage 8 showing the pollination drop. (C) Longitudinal section of a paraffin embedded bud with ovules at stage 4. (D) Longitudinal section of a paraffin embedded ovule at stage 8 in which the pollen chamber and the forming female gametophyte are visible. Fg, female gametophyte; L, leaf primordium; Ov, ovule primordium; Pc, pollen chamber.

In situ hybridization experiments were conducted on buds enclosing ovule and leaf primordia (Figure 1A, B) and on ovules during the pollination phase (Figure 1C, D), respectively stages 4 and 8 in D'Apice et al. (2021) (Figure 1). The study of these two time points in the ovule development highlights the changes of the expression domains of selected key regulatory genes during the early ovule development and during its subsequent maturation, which is represented by reaching the pollination phase.

**Figure 2:**
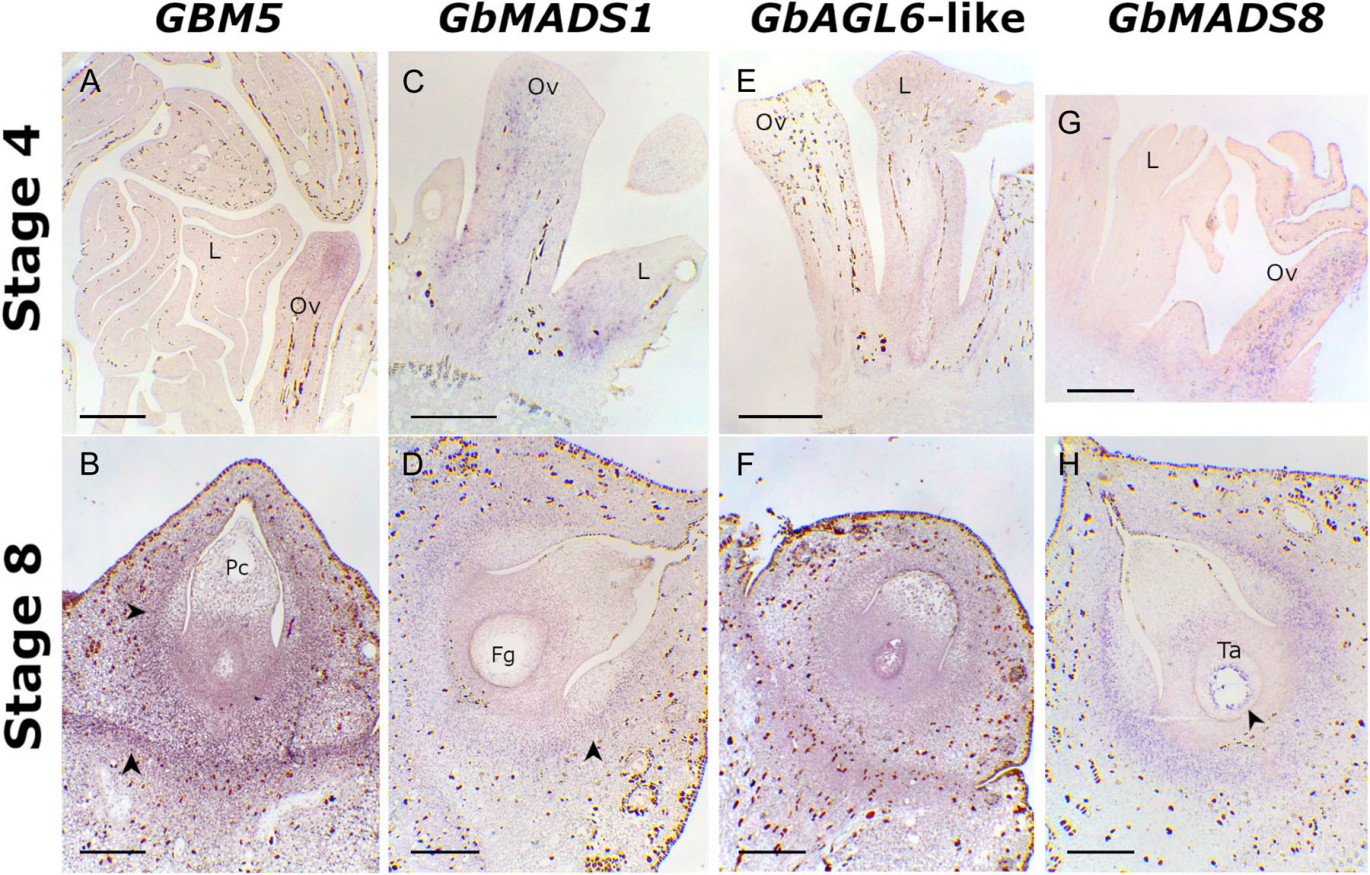
In situ hybridizations of *Ginkgo biloba GBM5*, *GbMADS1*, *GbAGL6*‐like and *GbMADS8* in ovules at stages 4 and 8 of development. (A) *GBM5* hybridization signal in ovule primordium (on the right) at stage 4. No signal detectable in leaf primordia. (B) *GBM5* hybridization signal in ovule at stage 8. Arrowheads indicate the region that will differentiate the sclerotesta, and the base of the ovule. (C) *GbMADS1* hybridization signal is visible in both ovule and leaf primordia. (D) *GbMADS1* hybridization signal in ovule at stage 8; the signal is stronger in the nucellus. Arrowhead indicates the region that will differentiate the sclerotesta. (E) *GbAGL6*‐like in leaf and ovule primordia at stage 4. The signal is faintly visible in differentiating vascular tissues. (F) *GbAGL6*‐like hybridization signal in ovule at stage 8; the signal is stronger in the nucellus and in the tapetum, but it is widespread throughout the ovule. (G) *GbMADS8* hybridization signal is visible in both reproductive and vegetative young structures, but mostly in the ovule. (H) *GbMADS8* hybridization signal in ovule at stage 8; the signal is stronger in the region that will differentiate the sclerotesta, and in the outermost part of the female gametophyte adjacent to the tapetum (black arrowhead). Fg, female gametophyte; L, leaf primordium; Ov, ovule primordium; Pc, pollen chamber; Ta, tapetum. Bars = 500 µm.

At stage 4 of ovule development, the expression of *GBM5*, the *Ginkgo* ortholog of *AG*, is detectable principally in the apical region of the ovule primordia; primarily, in the area in which the nucellus will differentiate from the integument (Figure 2A). Later, at stage 8 of ovule development, *GBM5* is expressed throughout the ovule, with the strongest signals coming from the base of the ovule and from around the nucellus, in the region of the integument that will differentiate into the sclerotesta (Figure 2B).

*GbMADS1, GbAGL6*‐like, and *GbMADS8* are the *Ginkgo AGL6* genes. Before bud opening (stage 4 of ovule development) the expression of *GbMADS1* (Figure 2C) is visible in the forming leaves and ovules, *GbAGL6*‐like is noticeable mainly in the differentiating vascular tissues of ovule and leaf primordia (Figure 2E) and *GbMADS8* is expressed only in ovule primordia (Figure 2G).

Interestingly, at pollination time *GbMADS1* (Figure 2D) and *GbAGL6*‐like (Figure 2F) showed a similar expression pattern. *GbMADS1* is noticeable throughout the integument, in the nucellus and in the contact area between the tapetum and the nucellar sporophytic tissue. *GbAGL6‐like* is also present in the whole integument and in the nucellus, but it is also visible in the tapetum encircling the female gametophyte. For both of these genes a weaker signal comes from the area at the base of the ovule. Differently from *GbMADS1* and *GbAGL6*‐like, *GbMADS8* expression is concentrated in the area of the ovule integument that will differentiate into the sclerotesta, and in the outermost part of the female gametophyte adjacent to the tapetum, including the membrane of the original functional megaspore (FM) (Figure 2H).

**Figure 3:**
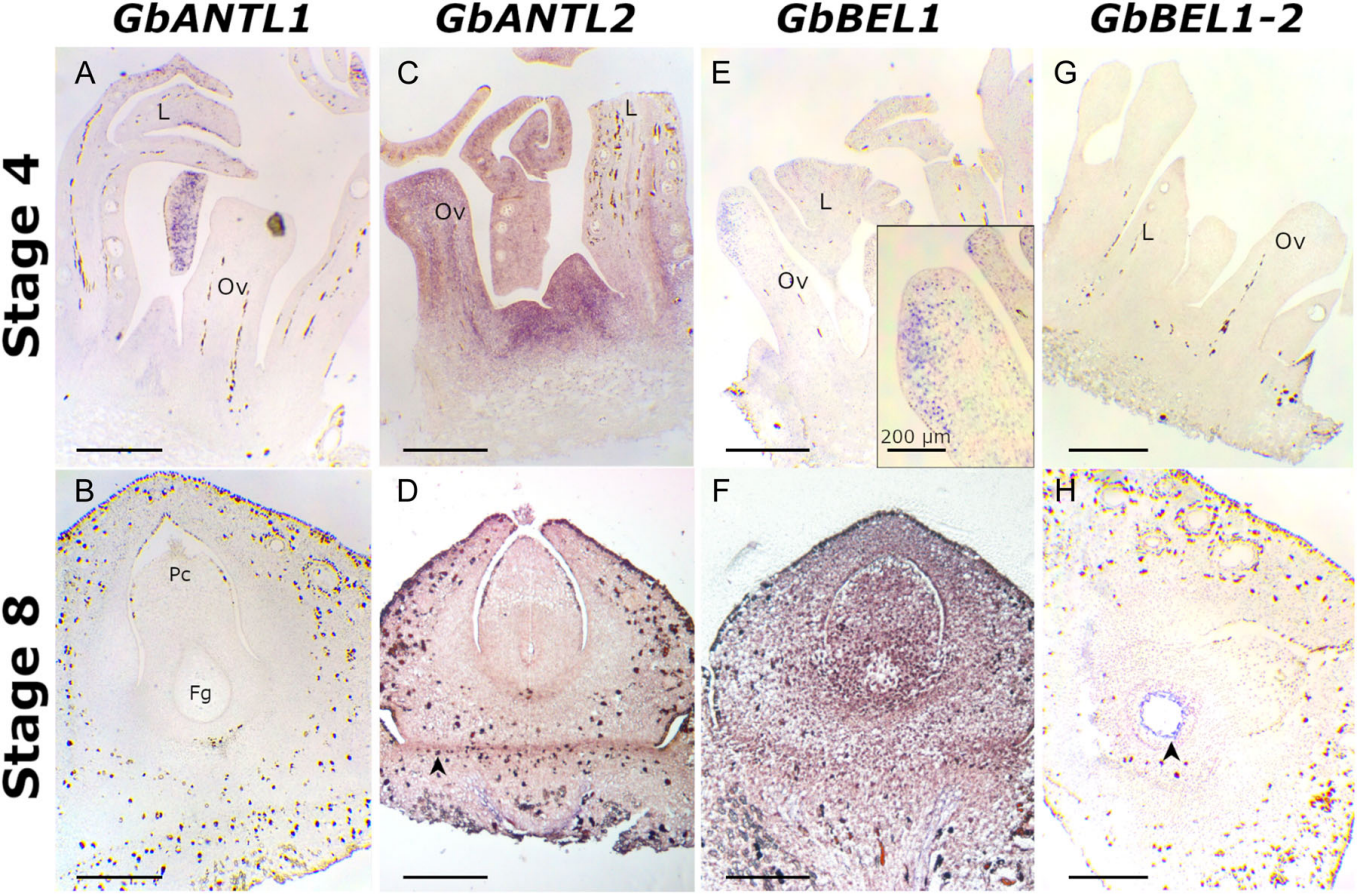
In situ hybridizations of *Ginkgo biloba GbANTL1*, *GbANTL2*, *GbBEL1*, *GbBEL1‐2* in ovules at stages 4 and 8 of development. (A) *GbANTL1* signal in leaf primordia. (B) *GbANTL1* is not detectable in ovule at stage 8. (C) *GbANTL2* hybridization signal in ovule and leaf primordia at stage 4. (D) *GbANTL2* hybridization signal in ovule at stage 8. Black arrowhead indicates the ovule abscission zone, where the signal is stronger. (E) *GbBEL1* signal in ovule at stage 4. The signal is clearly visible at the tip of the ovule, in the region that will differentiate the integument. (F) *GbBEL1* hybridization signal in ovule at stage 8. (G) *GbBEL1‐2* is not expressed in ovules at stage 4, neither in leaf primordia. (H) *GbBEL1‐2* signal in the portion of the female gametophyte that is adjacent to the tapetum in ovule (black arrowhead) at stage 8. Fg, female gametophyte; L, leaf primordium; Ov, ovule primordium; Pc, pollen chamber. Bars = 500 µm except where indicated differently.

*GbANTL1* and *GbANTL2* are two putative *Ginkgo* orthologous genes of the *ANT* gene. At stage 4 of ovule development, the signal for *GbANTL1* is visible only in young leaves (Figure 3A), while *GbANTL2* expression is detectable principally in differentiating and dividing tissues at the base of the buds, and throughout the ovule and leaf primordia (Figure 3C). Results obtained on ovules at stage 8 indicate that no signal is detectable for *GbANTL1* (Figure 3B), whereas *GbANTL2* expression becomes restricted at the base of the integument, where the stalk joins the ovule (Figure 3D).

With regards to *BEL1*, in this study we focused on two *Ginkgo* orthologs of *BEL1*: *GbBEL1* and *GbBEL1‐2*. The expression of *GbBEL1* is detectable in ovules at stage 4, with the strongest signal coming from the apex of the ovule, in the area where the nucellus and integument will differentiate (Figure 3E). In ovules at stage 8, at the moment of pollination, the expression of the gene is detectable throughout the ovule but most intensely in the nucellus (Figure 3F). *GbBEL1‐2* was expressed only at the time of pollination, mainly in the outermost region of the female gametophyte, adjacent to the tapetal sporophytic tissue of the ovule (Figure 3G, H).

**Figure 4:**
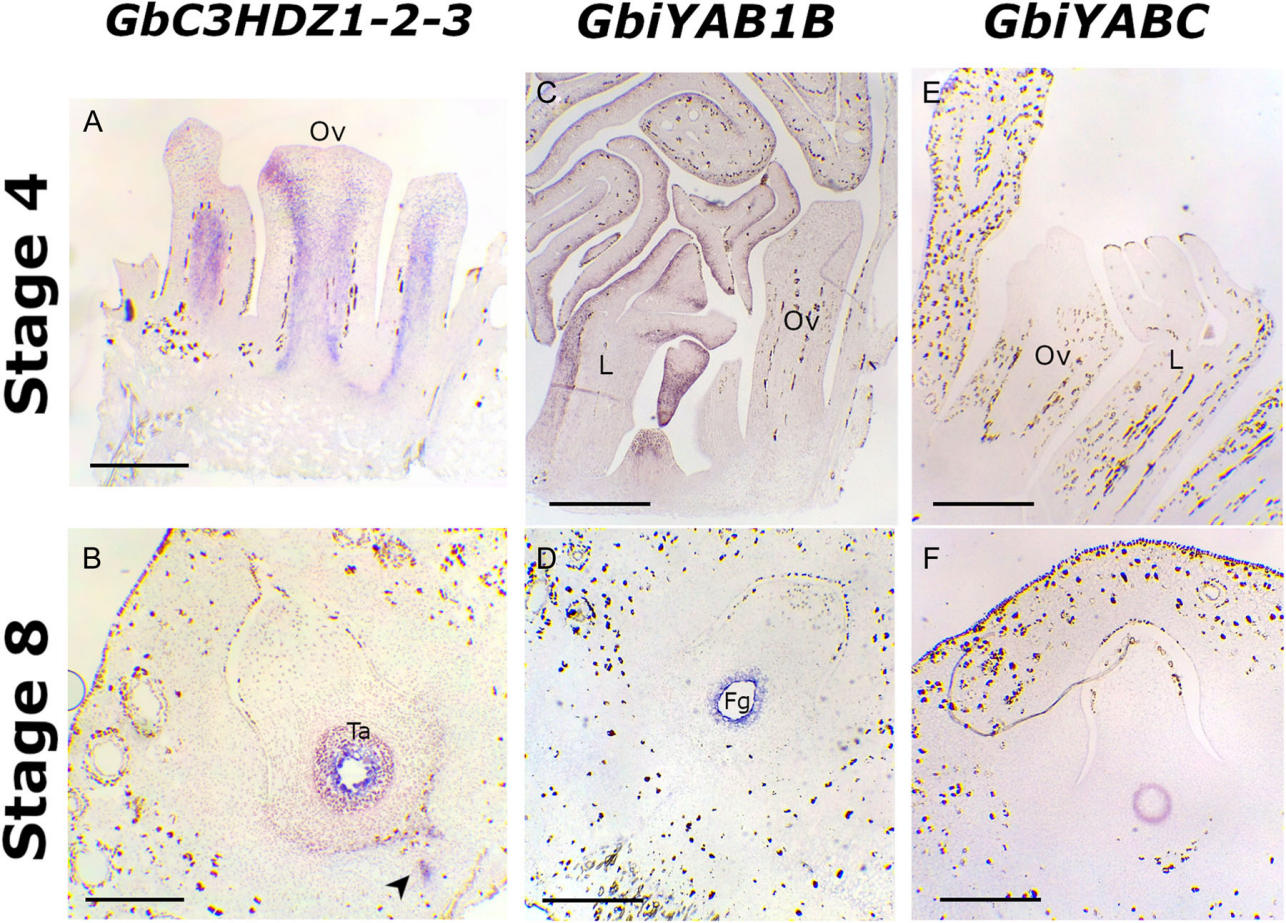
In situ hybridizations of *Ginkgo biloba GbiYAB1B* and *GbiYABC* in ovules at stages 4 and 8 of development. (A) *GbC3HDZ1‐2‐3* in differentiating vascular tissues of leaves and ovules at stage 4. (B) *GbC3HDZ1‐2‐3* signal in the tapetum, in the outermost portion of the female gametophyte, and in vascular tissues (black arrowhead) in ovule at stage 8. (C) *GbiYAB1B* signal detectable in the abaxial side of leaf primordia. (D) *GbiYAB1B* is detectable in the female gametophyte, and weakly in the tapetum in ovule at stage 8. (E) *GbiYABC* is not expressed in ovule and leaf primordia. (F) *GbiYABC* signal is visible in the tapetum in ovule at stage. Fg, female gametophyte; L, leaf primordium; Ov, ovule primordium; Ta, tapetum. Bars = 500 µm.

*Class III HD‐Zip* orthologs were studied using a probe; it hybridized with all three *C3HDZ1‐2‐3* genes. In buds, *C3HDZ1‐2‐3* are expressed in the developing vascular tissues of young leaves and ovules (Figure 4A), while at stage 8 their expression is detected in both sporophytic and gametophytic tissues. A strong signal comes from both the tapetum and the contact region of the latter with the female gametophyte. In addition, a signal is also present at the chalazal end of the nucellus and in the vascular tissues (Figure 4B).

**Figure 5:**
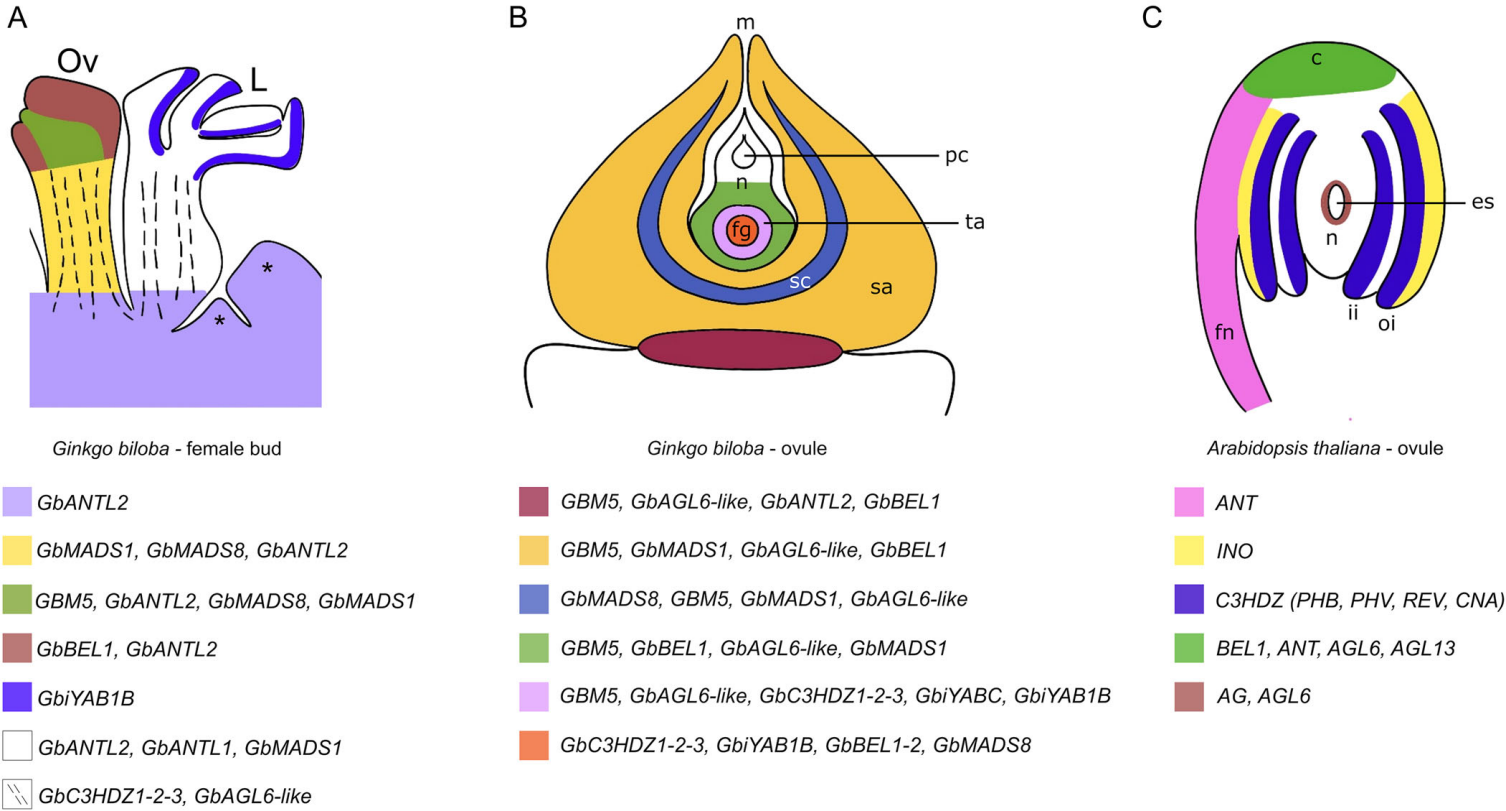
Schematic representation of the gene expression domains in *Ginkgo biloba* and *Arabidopsis thaliana* ovules. Expression domains are highlighted by different colors, and the genes whose expression has been detected in each domain are listed. (A) Gene expression domains of the studied genes in a *Gingko biloba* female bud. (B) Gene expression domains of the studied genes in a *Gingko biloba* ovule at pollination time. (C) Gene expression domains of *Arabidopsis* orthologs of the *Ginkgo* studied genes. c, chalaza; es, embryo sac; fg, female gametophyte; fn, funiculus; ii, inner integument; L, leaf primordium; m, micropyle; n, nucellus; oi, outer integument; Ov, ovule primordium; pc, pollen chamber; sa, sarcotesta; sc, sclerotesta; ta, tapetum; *, differentiating and dividing tissue.

Regarding *YABBY* orthologous genes, *GbiYAB1B* expression is not visible in the young ovule at stage 4, but a clear signal of expression has been observed on the abaxial side of all developing leaves (Figure 4C). At stage 8 of ovule development, the expression of *GbiYAB1B* is noticeable only in the outermost region of the female gametophyte, and less intensely in the tapetal sporophytic tissues (Figure 4D). *GbiYABC* expressed only at stage 8 of ovule development in the contact area between the tapetum and the nucellar sporophytic tissue (Figure 4E, F). A schematic model of gene expression is reported in Figure 5, in which a *Ginkgo* female bud and a *Ginkgo* ovule at pollination time are schematized (Figure 5A, B) for a complete overview of the obtained expression data. The gene expression domains of *Arabidopsis* orthologs of the *Ginkgo* studied genes are shown in Figure 5C.

## DISCUSSION

This study aimed to shed light on the genetic networks that regulate ovule development in *Ginkgo* by investigating the expression domains of *Ginkgo* orthologs of regulatory genes, which have been widely studied in model angiosperms. Recently, studies concerning the roles of these genes in nonmodel species and in nonflowering plants are arising (e.g., Floyd et al., 2006; Finet et al., 2016).

Experiments have been designed to investigate two stages of *Ginkgo* ovule development: early ovules still contained inside wintering buds, and ovules at the pollination time. Early ovules (stage 4 of ovule development from D'Apice et al., 2021) display a still undifferentiated and incomplete integument—because it has not yet completely encircled the underneath nucellus—therefore representing a very early stage of development that has not been characterized so far in terms of gene expression patterns. Because most of the genes we have taken into consideration are required both for the determination and the emergence of the ovule primordia and for the integument morphogenesis in the model plant *Arabidopsis thaliana*, we wanted to understand if also in *Ginkgo* the expression of these orthologs is precocious as in *Arabidopsis*. The second stage analyzed in this study is the pollination stage (stage 8 of ovule development from D'Apice et al., 2021). At this stage, ovules display an integument that has completely encircled the nucellus with a completely formed micropyle; however, the three characteristic layers in which the single integument will differentiate to form the seed coat are not yet recognizable. Therefore, stage 8 is still an early stage of development (albeit advanced in respect to stage 4) because the female gametophyte has just started its mitotic divisions that four months later will form the mature female gametophyte (D'Apice et al., 2021). By studying these two time points, we have been able to obtain an overview of the changes that occur inside the buds and therefore in the developing young ovules for the selected regulatory genes. Moreover, here we performed in situ hybridization experiments on buds showing both ovule and leaf primordia, whereas most of the expression studies conducted so far for *Ginkgo biloba* were performed on ovules more advanced in their development.

*AGAMOUS* is a MICK^C^ type MADS‐box gene widely known to be involved in ovule identity determination and ovule development (Ray et al., 1994; Western and Haughn, 1999; Pinyopich et al., 2003; Gramzow and Theissen, 2010). The *AG* expression becomes restricted to the endothelium that surrounds the embryo sac in fully differentiated carpels (Bowman et al., 1991). In *Arabidopsis*, *AG* together with *SEPALLATA* (*SEP*) genes, is also required for determining the carpel identity (Thomson and Wellmer, 2019; Barro‐Trastoy et al., 2020). *AGAMOUS‐like6* (*AGL6*) and *SEP* genes form two sister clades, with *SEP* being exclusive of angiosperms (Zahn et al., 2005). *Arabidopsis AGL6* paralogs are expressed in the endothelium and in the chalazal end of the ovule (Schauer et al., 2009) (Figure 5C).

In this study, we have characterized the expression patterns of the *Ginkgo AG* and *AGL6* genes, confirming that *GBM5*, *GbMADS1*, and *GbMADS8* are expressed throughout the ovule at pollination time, which are consistent with results obtained by Lovisetto et al. (2012). We also found that *GbAGL6*‐like is strongly expressed in ovules at stage 8. Interestingly, *GbMADS8* expression is strong in the female gametophyte, suggesting that it could have a role during the development of the female gametophyte. The other MADS‐box genes (*GBM5*, *GbMADS1*, and *GbAGL6‐like*) are mostly expressed, and therefore probably involved, in the development of the sporophytic tissue, because their expression remains confined outside the membrane of the original functional megaspore. Moreover, the signal of *GBM5* was stronger compared to the signals of the three *AGL6* genes. In the buds (stage 4), *GBM5* is expressed exclusively in the ovule, confirming what was previously observed by Chen et al. (2017). However, the absence of *AG* expression in young vegetative tissues (such as young *Ginkgo* leaves) disagrees with what was previously suggested by Jager et al. (2003), even though we cannot exclude a low constitutive expression level in these structures, which are not detectable with in situ hybridizations. Consistently, as in Chen et al. (2017), *GbMADS1* is weakly expressed in both vegetative and reproductive structures in buds, sharing a similar expression pattern with *GbAGL6*‐like. Interestingly, *GbMADS8* seems to be specifically required for the early ovule development, because it is not expressed in leaf primordia. Our expression analyses of MADS‐box genes confirm the essential role of *GBM5* for the proper ovule development from its emergence to its differentiation, and also suggests that *GbMADS8* has an important role during ovule development, because it is expressed at both of the stages taken into account in this study. In contrast, the other two *AGL6* orthologs (*GbMADS1* and *GbAGL6*‐like) seem to be involved later in ovule development. Indeed, their expression levels increase in ovules at stage 8, suggesting a certain grade of redundancy for *GbMADS1* and *GbAGL6*‐like in their expression pattern during ovule development (particulary in the sporophytic tissues: the integument and the nucellus). Instead, *GbMADS8* might be more specialized in driving the female gametophyte development.

Regarding the state of the art in *Arabidopsis*, it is widely known that the *AINTEGUMENTA* gene encodes for a two‐AP2domain‐containing transcription factor required for ovule primordia initiation and development because it promotes cellular divisions during the early stage of ovule development in *Arabidopsis* (Elliot et al., 1996; Klucher et al., 1996; Krizek, 1999; Cucinotta et al., 2014; Barro‐Trastoy et al., 2020). *ANT* is required for embryo sac maturation and for the proper formation of the integuments (Elliot et al., 1996; Klucher et al., 1996), together with the homeodomain gene *BELL1* (*BEL1*), which specifically marks the chalazal region—known to be required for inner integument development and outer integument identity (Robinson‐Beers et al., 1992; Reiser et al., 1995; reviewed in Barro‐Trastoy et al., 2020). During last decades *ANT* and *BEL1* genes were also being investigated in nonmodel angiosperms and gymnosperms (i.e., in *Gnetum gnemon* in Becker et al., 2002). In situ hybridizations conducted on *Pinus thunbergia* (Shigyo and Ito, 2004) and *Gnetum parvifolium* ovules (Yamada et al., 2008) suggest that the respective homologs *PtANTL1* and *GpANTL1* have an important role in ovule development. Shigyo and Ito (2004) suggest that *PtANTL1* has the same role as *ANT* in controlling lateral organ size and lateral organ development. Accordingly, Yamada et al. (2008) evidenced that the pattern of expression of *GpANTL1* in the *G. parvifolium* ovular envelopes and leaves is consistent with that of *ANT* in *Arabidopsis*, speculating that this could be the ancestral expression pattern of the *ANT* (s.s.) clade (Yamada et al., 2008). Our results agree with these observations. We found *GbANTL2* expressed in the actively dividing tissues at the base of the bud and in both leaf and ovule primordia. Moreover, the expression of the *Ginkgo* homolog decreases and it localizes at the base of the ovule, in the abscission zone, during later stages of development, as observed also by Zumajo‐Cardona et al. (2021). A decreasing expression of *GbANTL2* in *Ginkgo* during ovule development can be comparable to the observed decreased expression of *GpANTL1* in developed ovules of *G. parvifolium* (Yamada et al., 2008). In contrast to *Arabidopsis ANT*, the gymnosperm orthologs *PtANTL1* and *GpANTL1* are expressed also in the nucellar tip of the ovule (Shigyo and Ito, 2004; Yamada et al., 2008). Hence, the expression pattern of the gymnosperm *ANT* in the ovulate axes can be considered a conserved trait, and this hypothesis is corroborated by our observations in *Ginkgo*. Different from *GbANTL2*, *GbANTL1* resulted as weakly expressed in buds and slightly more marked in leaves, whereas it is not detectable in ovules at pollination contrary to what was observed for *GbANTL2*. We hypothesize that *GbANTL2* controls the ovule development in both early and later stages of development, suggesting that *GbANTL2* and angiosperm *ANT* genes are functional homologs. Whereas *GbANTL1* seems to be not involved in ovule development, these differences in the expression patterns of the two genes therefore suggest a putative subfunctionalization or neofunctionalization after gene duplication. *ANT* gene duplication, which has occurred in gymnosperms, is also supported by our phylogenetic analysis.

Even though *BEL1* was one of the first genes identified as an ovule regulatory gene in *Arabidopsis* (Ray et al., 1994; Western and Haughn, 1999; Rudall, 2021), very few studies regarding *BEL1* orthologs in gymnosperms are available so far. The first *BEL1*‐like genes (*MELBEL1*‐*MELBEL4*) of a nonflowering plant were studied by Becker et al. (2002) in the gymnosperm *Gnetum gnemon*. The most recent study revealed that in young ovules of *G. gnemon*, *MELBEL1* is expressed in the nucellus, while in more mature ovules it remains expressed in the nucellus and in the megaspores (after meiosis), but no expression is detected in the pollen chamber, in either the integument or envelopes (Zumajo‐Cardona and Ambrose, 2021). Our results have shown that in *Ginkgo*, the two orthologs have a differentiated temporal expression pattern, suggesting that *GbBEL1* has a role in both stages. The second ortholog *GbBEL1‐2* is activated only later during the ovule development and is required in the female gametophyte development. Our results regarding *GbBEL1* expression are in accordance with what observed by Zumajo‐Cardona et al. (2021) for the same gene. The expression of *GbBEL1‐2* seems to be complementary to *GbBEL1* at pollination time in that it is expressed only in the developing female gametophyte while *GbBEL1* is detectable throughout nucellus and integument. More studies are required to elucidate the expression pattern of these several *BEL1*‐like genes in *Ginkgo biloba*. The few data available for expression domains of *BEL1*‐like genes in gymnosperm developing ovules could lead to a hypothesis that the roles of these genes in nonflowering plants concern ovule determination and patterning. In contrast to *Arabidopsis BEL1*, they are probably mostly involved in the female gametophyte development, at least in *Ginkgo* and *Gnetum gnemon* (Zumajo‐Cardona et al., 2021; this study).

In *Arabidopsis*, once the identity of the integuments is established, the genes required for integument morphogenesis come into play. In particular, the regulators of abaxial and adaxial polarity of the integument such as INO and Class III HD‐ZIP transcription factors are recruited (Villanueva et al., 1999; Kelley et al., 2009; Barro‐Trastoy et al., 2020). Our study regarding the role of the *Ginkgo YABBY* orthologs finally confirmed the expression of *GbiYAB1B* in the abaxial side of leaf primordia, which is consistent with Finet et al. (2016). This expression pattern in leaf primordia can be compared to the expression pattern of the *YABBY* genes in angiosperms, which are expressed in the abaxial side of lateral organs, possibly representing the ancestral expression pattern for the *YABBY* gene family (Finet et al., 2016). However, our results have shown that *GbiYABC* is not expressed in the abaxial side of young leaves, suggesting that this gene could be recruited later in the development of leaves. *GbiYABC* seems also not required for the development of young ovule primordia. It is recruited later during the ovule development because it is expressed in the tapetum, while at the same stage, *GbiYAB1B* is expressed in the outermost part of the female gametophyte, suggesting a functional differentiation of these two *YABBY* genes.

The hybridization signal of the three *Class III HD‐ZIP Ginkgo* genes highlighted a putative role of them in provasculatures of both leaf and ovule primordia, as also observed by Floyd et al. (2006), and in the vasculatures of mature ovules. Strikingly, their expression domain in mature ovules moves to the tapetal cells encircling the female gametophyte and to the contact layer of the female gametophyte with the sporophytic tapetal tissue. The circumscribed and concomitant expression of several regulatory genes, for instance *GbBEL1‐2*, *GbC3HDZ*, and *GbiYAB1B* and *GbiYABC* in the contact area between the sporophyte and the female gametophyte, could suggest that in *Ginkgo* the sporophyte also plays a crucial role in promoting the synchronized female gametophyte development, as it is in *Arabidopsis* (Bencivenga et al., 2011; Figueiredo and Köhler, 2016). Indeed, it has been demonstrated that in *Arabidopsis* the sporophytic mutants for some of the above cited genes show defects in the gametophyte development (Bencivenga et al., 2011; Figueiredo and Köhler, 2016). During our sampling activity, we frequently observed *Ginkgo* ovules showing defects during their development. Generally, in these ovules the integument appears correctly developed, while internally the female gametophyte is degenerated. Therefore, the major players acting in sporophytic maternal tissue that are involved in the regulation of the gametophyte development might also be acting in other nonflowering plants. The schematic representation of the gene expression domains reported in Figure 5 suggests a complex dialogue between the sporophyte and the gametophyte during ovule development. Furthermore, by comparing *Ginkgo* and *Arabidopsis* (Figure 5) it was possible to observe that some of the genes studied in this work showed a more widespread expression domain in the *Ginkgo* ovule, in contrast to the more delimited domains that characterize the ovule of *Arabidopsis*. A possible explanation could be the different development time of the ovule in the two plants, which is much longer in *Ginkgo*. Therefore, a spatiotemporal modification of gene expression in the *Ginkgo* ovules could take longer, comparing to *Arabidopsis*. Further studies are needed to elucidate the genes involved in the crosstalk between the sporophyte and the gametophyte, and in investigating ovule development in other gymnosperm species.

## CONCLUSIONS

The study of ovule development regulatory genes in nonmodel species is important to understand the functional evolution of the genetic network of ovule development in seed plants and its effect on the morphological evolution of land plant reproductive structures. Our work aimed to study the spatiotemporal expression of regulatory genes in two stages of ovule development in *Ginkgo biloba* through in situ hybridization experiments. Overall, our results indicate that certain genes (e.g., *Ginkgo AG*, *AGL6*, and *C3HDZ1‐2‐3*) are expressed in a way that is comparable to how their orthologs are expressed in *Arabidopsis* and/or other gymnosperms, suggesting a conserved function among seed plants. Other genes (e.g., the *Ginkgo ANT*, *BELL1*, and *YABBY*), display patterns of expression only partially comparable to those of other seed plants, with peculiarities that to our current knowledge, are unique to *Ginkgo*. The study of these regulatory genes could be difficult and puzzling in species that are poorly studied. For this reason, the investigation of ovule development and its regulation needs to be extended to many other plant species.

## AUTHOR CONTRIBUTIONS

B.B., L.B., and S.M. conceived the study; S.M. and G.D'.A. designed the experiments; G.D'.A., S.M., R.C., A.M., and S.N. carried out the experiments; G.D'.A. and S.M. wrote the first draft; B.B., L.B., and S.N. revised the manuscript; all authors commented on the manuscript and agreed with the submission.

## Supporting information


**Appendix S1**. Forward (F) and reverse (R) primers used to synthetize the probes for in situ hybridization experiments.

**Appendix S2**. Data set for the phylogenetic analysis of YABBY protein sequences.

**Appendix S3**. Data set for the phylogenetic analysis of AINTEGUMENTA protein sequences.

**Appendix S4**. Maximum Likelihood (ML) phylogenetic analysis of YABBY protein sequences. 1000 bootstrap replicates, cut‐off of bootstrap values in the figure is 70%. Highlighted with colors from the top to the bottom, the CRABS CLAW (CRC) group, the YAB5 group, a well‐supported group of YABBY proteins of gymnosperms that contains GbiYAB1B, the INO group and the other group of gymnosperm YABBY sequences, which contains GbiYABC and GbiYABA.

**Appendix S5**. Maximum Likelihood (ML) phylogenetic analysis of AINTEGUMENTA protein sequences; 1000 bootstrap replicates, cut‐off of bootstrap values in the figure is 70%. Highlighted with colors are the two well‐divided groups of ANT sequences of gymnosperms. (A) The group containing GbANTL2, and (B) the group with GbANTL1.

## Data Availability

Primer sequences used to amplify the gene fragments for synthesis of the probes are provided in Appendix S1. Phylogenetic information of *YABBY* and *AINTEGUMENTA* gene clades are available in Appendices S2, S3, S4, and S5.

## References

[ajb21862-bib-0001] Ambrose, B. A. , D. R. Lerner , P. Ciceri , C. M. Padilla , M. F. Yanofsky , and R. J. Schmidt . 2000. Molecular and genetic analyses of the *silky1* gene reveal conservation in floral organ specification between eudicots and monocots. Molecular Cell 5: 569–579. 10882141 10.1016/s1097-2765(00)80450-5

[ajb21862-bib-0002] Barro‐Trastoy, D. , M. Dolores Gomez , P. Tornero , and M. A. Perez‐Amador . 2020. On the way to ovules: The hormonal regulation of ovule development. Critical Reviews in Plant Sciences 39: 431–456.

[ajb21862-bib-0003] Becker, A. , M. Bey , T. R. Bürglin , H. Saedler , and G. Theissen . 2002. Ancestry and diversity of BEL1‐like homeobox genes revealed by gymnosperm (*Gnetum gnemon*) homologs. Development Genes and Evolution 212: 452–457. 12373591 10.1007/s00427-002-0259-7

[ajb21862-bib-0004] Bencivenga, S. , L. Colombo , and S. Masiero . 2011. Cross talk between the sporophyte and the megagametophyte during ovule development. Sexual Plant Reproduction 24: 113–121. 21298290 10.1007/s00497-011-0162-3

[ajb21862-bib-0005] Bowman, J. L. , G. N. Drews , and E. M. Meyerowitz . 1991. Expression of the Arabidopsis floral homeotic gene *AGAMOUS* is restricted to specific cell types late in flower development. The Plant Cell 3: 749–758. 1726485 10.1105/tpc.3.8.749 PMC160042

[ajb21862-bib-0006] Carlsbecker, A. , K. Tandre , U. Johanson , M. Englund , and P. Engström . 2004. The MADS‐box gene *DAL1* is a potential mediator of the juvenile‐to‐adult transition in Norway spruce (*Picea abies*). The Plant Journal 40: 546–557. 15500470 10.1111/j.1365-313X.2004.02226.x

[ajb21862-bib-0007] Chang, S. , J. Puryear , and J. Cairney . 1993. A simple and efficient method for isolating RNA from pine trees. Plant Molecular Biology Reporter 11: 113–116.

[ajb21862-bib-0008] Chen, F. , X. Zhang , X. Liu , and L. Zhang . 2017. Evolutionary analysis of MIKC^C^‐type MADS‐box genes in gymnosperms and angiosperms. Frontiers in Plant Science 8: 895. 28611810 10.3389/fpls.2017.00895 PMC5447709

[ajb21862-bib-0010] Cucinotta, M. , L. Colombo , and I. Roig‐Villanova . 2014. Ovule development, a new model for lateral organ formation. Frontiers in Plant Science 5: 117. 24723934 10.3389/fpls.2014.00117 PMC3973900

[ajb21862-bib-0011] Cucinotta, M. , M. Di Marzo , A. Guazzotti , S. de Folter , M. M. Kater , and L. Colombo . 2020. Gynoecium size and ovule number are interconnected traits that impact seed yield. Journal of Experimental Botany 71: 2479–2489. 32067041 10.1093/jxb/eraa050 PMC7210752

[ajb21862-bib-0012] D'Apice, G. , S. Moschin , F. Araniti , S. Nigris , M. Di Marzo , A. Muto , C. Banfi , et al. 2021. The role of pollination in controlling *Ginkgo biloba* ovule development. New Phytologist 232: 2353–2368. 34558676 10.1111/nph.17753 PMC9292720

[ajb21862-bib-0013] Darriba, D. , D. Posada , A. M. Kozlov , A. Stamatakis , B. Morel , and T. Flouri . 2020. ModelTest‐NG: a new and scalable tool for the selection of DNA and protein evolutionary models. Molecular Biology and Evolution 37: 291–294. 31432070 10.1093/molbev/msz189 PMC6984357

[ajb21862-bib-0014] Darriba, D. , G. L. Taboada , R. Doallo , and D. Posada . 2012. jModelTest 2: more models, new heuristics and parallel computing. Nature Methods 9: 772–772. 10.1038/nmeth.2109 PMC4594756 22847109

[ajb21862-bib-0015] Douglas, A. W. , D. W. Stevenson , and D. P. Little . 2007. Ovule development in *Ginkgo biloba* L., with emphasis on the collar and nucellus. International Journal of Plant Sciences 168: 1207–1236.

[ajb21862-bib-0016] Elliott, R. C. , A. S. Betzner , E. Huttner , M. P. Oakes , W. Q. Tucker , D. Gerentes , P. Perez , and D. R. Smyth . 1996. *AINTEGUMENTA*, an *APETALA2*‐like gene of *Arabidopsis* with pleiotropic roles in ovule development and floral organ growth. The Plant Cell 8: 155–168. 8742707 10.1105/tpc.8.2.155 PMC161088

[ajb21862-bib-0017] Eshed, Y. , S. F. Baum , J. V. Perea , and J. L. Bowman . 2001. Establishment of polarity in lateral organs of plants. Current Biology 11: 1251–1260. 11525739 10.1016/s0960-9822(01)00392-x

[ajb21862-bib-0018] Figueiredo, D. D. , and C. Köhler . 2016. Bridging the generation gap: communication between maternal sporophyte, female gametophyte and fertilization products. Current Opinion in Plant Biology 29: 16–20. 26658334 10.1016/j.pbi.2015.10.008

[ajb21862-bib-0019] Finet, C. , S. K. Floyd , S. J. Conway , B. Zhong , C. P. Scutt , and J. L. Bowman . 2016. Evolution of the *YABBY* gene family in seed plants. Evolution and Development 18: 116–126. 26763689 10.1111/ede.12173

[ajb21862-bib-0020] Floyd, S. K. , C. S. Zalewski , and J. L. Bowman . 2006. Evolution of class III homeodomain–leucine zipper genes in streptophytes. Genetics 173: 373–388. 16489224 10.1534/genetics.105.054239 PMC1461458

[ajb21862-bib-0021] Gasser, C. S. , and D. J. Skinner . 2019. Development and evolution of the unique ovules of flowering plants. Current Topics in Developmental Biology 131: 373–399. 30612624 10.1016/bs.ctdb.2018.10.007

[ajb21862-bib-0023] Gramzow, L. , and G. Theissen . 2010. A hitchhiker's guide to the MADS world of plants. Genome Biology 11: 1–11 10.1186/gb-2010-11-6-214 PMC2911102 20587009

[ajb21862-bib-0024] Guan, R. , Y. Zhao , H. Zhang , G. Fan , X. Liu , W. Zhou , C. Shi , et al. 2016. Draft genome of the living fossil *Ginkgo biloba* . Gigascience 5: 13742–016 10.1186/s13742-016-0154-1 PMC5118899 27871309

[ajb21862-bib-0026] Jager, M. , A. Hassanin , M. Manuel , H. L. Guyader , and J. Deutsch . 2003. MADS‐box genes in *Ginkgo biloba* and the evolution of the *AGAMOUS* family. Molecular Biology and Evolution 20: 842–854. 12679535 10.1093/molbev/msg089

[ajb21862-bib-0027] Katoh, K. , J. Rozewicki , and K. D. Yamada . 2019. MAFFT online service: multiple sequence alignment, interactive sequence choice and visualization. Briefings in Bioinformatics 20: 1160–1166. 28968734 10.1093/bib/bbx108 PMC6781576

[ajb21862-bib-0028] Kelley, D. R. , and C. S. Gasser . 2009. Ovule development: genetic trends and evolutionary considerations. Sexual Plant Reproduction 22: 229–234. 20033444 10.1007/s00497-009-0107-2 PMC2796119

[ajb21862-bib-0029] Kelley, D. R. , D. J. Skinner , and C. S. Gasser . 2009. Roles of polarity determinants in ovule development. The Plant Journal 57: 1054–1064. 19054366 10.1111/j.1365-313X.2008.03752.x PMC4096117

[ajb21862-bib-0030] Klucher, K. M. , H. Chow , L. Reiser , and R. L. Fischer . 1996. The *AINTEGUMENTA* gene of *Arabidopsis* required for ovule and female gametophyte development is related to the floral homeotic gene *APETALA2* . Plant Cell 8: 137–153. 8742706 10.1105/tpc.8.2.137 PMC161087

[ajb21862-bib-0031] Krizek, B. A. 1999. Ectopic expression of *AINTEGUMENTA* in *Arabidopsis* plants results in increased growth of floral organs. Developmental Genetics 25: 224–236. 10528263 10.1002/(SICI)1520-6408(1999)25:3<224::AID-DVG5>3.0.CO;2-Y

[ajb21862-bib-0032] Lovisetto, A. , F. Guzzo , A. Tadiello , K. Toffali , A. Favretto , and G. Casadoro . 2012. Molecular analyses of MADS‐box genes trace back to gymnosperms the invention of fleshy fruits. Molecular Biology and Evolution 29: 409–419. 21972256 10.1093/molbev/msr244

[ajb21862-bib-0033] McAbee, J. M. , T. A. Hill , D. J. Skinner , A. Izhaki , B. A. Hauser , R. J. Meister , G. V. Reddy , et al. 2006. *ABERRANT TESTA SHAPE* encodes a KANADI family member, linking polarity determination to separation and growth of *Arabidopsis* ovule integuments. The Plant Journal 46: 522–531. 16623911 10.1111/j.1365-313X.2006.02717.x

[ajb21862-bib-0034] Mizukami, Y. , and R. L. Fischer . 2000. Plant organ size control: *AINTEGUMENTA* regulates growth and cell numbers during organogenesis. Proceedings of the National Academy of Sciences 97: 942–947. 10.1073/pnas.97.2.942 PMC15435 10639184

[ajb21862-bib-0036] Pinyopich, A. , G. S. Ditta , B. Savidge , S. J. Liljegren , E. Baumann , E. Wisman , and M. F. Yanofsky . 2003. Assessing the redundancy of MADS‐box genes during carpel and ovule development. Nature 424: 85–88. 12840762 10.1038/nature01741

[ajb21862-bib-0037] Posada, D. 2008. jModelTest: phylogenetic model averaging. Molecular Biology and Evolution 25: 1253–1256. 18397919 10.1093/molbev/msn083

[ajb21862-bib-0038] Ray, A. , K. Robinson‐Beers , S. Ray , S. C. Baker , J. D. Lang , D. Preuss , S. B. Milligan , and C. Gasser . 1994. *Arabidopsis* floral homeotic gene *BELL* (*BEL1*) controls ovule development through negative regulation of *AGAMOUS* gene (*AG*). Proceedings of the National Academy of Sciences 91: 5761–5765. 10.1073/pnas.91.13.5761 PMC44076 7912435

[ajb21862-bib-0039] Reiser, L. , Z. Modrusan , L. Margossian , A. Samach , N. Ohad , G. W. Haughn , and R. L. Fischer . 1995. The *BELL1* gene encodes a homeodomain protein involved in pattern formation in the *Arabidopsis* ovule primordium. Cell 83: 735–742. 8521490 10.1016/0092-8674(95)90186-8

[ajb21862-bib-0040] Robinson‐Beers, K. , R. E. Pruitt , and C. S. Gasser . 1992. Ovule development in wild‐type *Arabidopsis* and two female‐sterile mutants. The Plant Cell 4: 1237–1249. 12297633 10.1105/tpc.4.10.1237 PMC160211

[ajb21862-bib-0042] Rudall, P. J. 2021. Evolution and patterning of the ovule in seed plants. Biological Reviews 96: 943–960. 33432779 10.1111/brv.12684

[ajb21862-bib-0043] Schauer, S. E. , S. E. Jacobsen , D. W. Meinke , and A. Ray . 2002. *DICER‐LIKE1*: blind men and elephants in *Arabidopsis* development. Trends in Plant Science 7: 487–491. 12417148 10.1016/s1360-1385(02)02355-5

[ajb21862-bib-0044] Schauer, S. E. , P. M. Schlüter , R. Baskar , J. Gheyselinck , A. Bolaños , M. D. Curtis , and U. Grossniklaus . 2009. Intronic regulatory elements determine the divergent expression patterns of *AGAMOUS‐LIKE6* subfamily members in *Arabidopsis* . The Plant Journal 59: 987–1000. 19473325 10.1111/j.1365-313X.2009.03928.x

[ajb21862-bib-0045] Shigyo, M. , M. Hasebe , and M. Ito . 2006. Molecular evolution of the AP2 subfamily. Gene 366: 256–265. 16388920 10.1016/j.gene.2005.08.009

[ajb21862-bib-0046] Shigyo, M. , and M. Ito . 2004. Analysis of gymnosperm two‐AP2‐domain‐containing genes. Development Genes and Evolution 214: 105–114. 14986134 10.1007/s00427-004-0385-5

[ajb21862-bib-0049] Thomson, B. , and F. Wellmer . 2019. Molecular regulation of flower development. Current Topics in Developmental Biology 131: 185–210. 30612617 10.1016/bs.ctdb.2018.11.007

[ajb21862-bib-0050] Villanueva, J. M. , J. Broadhvest , B. A. Hauser , R. J. Meister , K. Schneitz , and C. S. Gasser . 1999. *INNER NO OUTER* regulates abaxial‐adaxial patterning in Arabidopsis ovules. Genes and Development 13: 3160–3169. 10601041 10.1101/gad.13.23.3160 PMC317184

[ajb21862-bib-0051] Wan, Y. , J. B. Ye , G. Wang , W. Zhang , F. Xu , and Y. Liao . 2016. Molecular cloning and sequence analysis of MADS‐box family gene (*GbMADS6*) from *Ginkgo biloba* L. International Journal of Current Research in Biosciences and Plant Biology 3: 73–79.

[ajb21862-bib-0052] Wang, L. , Z. Lu , W. Li , J. Xu , K. Luo , W. Lu , L. Zhang , and B. Jin . 2016. Global comparative analysis of expressed genes in ovules and leaves of *Ginkgo biloba* L. Tree Genetics and Genomes 12: 1–18.

[ajb21862-bib-0053] Western, T. L. , and G. W. Haughn . 1999. *BELL1* and *AGAMOUS* genes promote ovule identity in *Arabidopsis thaliana* . The Plant Journal 18: 329–336. 10377998 10.1046/j.1365-313x.1999.00448.x

[ajb21862-bib-0054] Yamada, T. , Y. Hirayama , R. Imaichi , and M. Kato . 2008. *AINTEGUMENTA* homolog expression in *Gnetum* (Gymnosperms) and implications for the evolution of ovulate axes in seed plants. Evolution & Development 10: 280–287. 18460090 10.1111/j.1525-142X.2008.00237.x

[ajb21862-bib-0055] Zahn, L. M. , H. Kong , J. H. Leebens‐Mack , S. Kim , P. S. Soltis , L. L. Landherr , D. E Soltis , et al. 2005. The evolution of the *SEPALLATA* subfamily of MADS‐box genes: a preangiosperm origin with multiple duplications throughout angiosperm history. Genetics 169: 2209–2223. 15687268 10.1534/genetics.104.037770 PMC1449606

[ajb21862-bib-0056] Zumajo‐Cardona, C. , and B. A. Ambrose . 2021. Deciphering the evolution of the ovule genetic network through expression analyses in *Gnetum gnemon* . Annals of Botany 128: 217–230. 33959756 10.1093/aob/mcab059 PMC8324035

[ajb21862-bib-0057] Zumajo‐Cardona, C. , D. P. Little , D. Stevenson , and B. A. Ambrose . 2021. Expression analyses in *Ginkgo biloba* provide new insights into the evolution and development of the seed. Scientific Reports 11: 1–17. 34754044 10.1038/s41598-021-01483-0 PMC8578549

