# Supplementary material for: Identification of key regulatory genes involved in the sporophyte and gametophyte development in Ginkgo biloba ovules revealed by in situ expression analyses
Source: Am J Bot. 2022 May 19;109(6):887–98. doi: 10.1002/ajb2.1862 (PMC9322462; doi:10.1002/ajb2.1862)
Supplement: Supplementary file 1 — Appendix S1. Forward (F) and reverse (R) primers used to synthetize the probes for in situ hybridization experiments. [file AJB2-109-887-s002.pdf]

Appendix S1: Forward (F) and reverse (R) primers used to synthetize the probes for *in situ* hybridization experiments.

**Gene name**	***Ginkgo* CDS code from Guan et al. (2016)**		**Primer sequence**
GbANTL1	Gb_05487	F	GACCAGGGCCATCCTTTTCA
		R	CAGATGCTGCAGCAGTTGGA
GbANTL2	Gb_07049	F	TGGGTTGAGCATGATGAGGA
		R	TGATCATCTGCATGCCAACG
GbBEL1	Gb_36166	F	GCCATCACAGCAAAGGACAT
		R	GGGCAGTAGGAAGCGGAATA
GbBEL1-3	Gb_39741	F	ACAGATACAGCGGCGTTTTC
		R	AGGTTCCTGACTCCACTGTG
GBM5	Gb_16301	F	CTCAGGGTGGAGCCATTTCA
		R	GGCCGTTTGATCCTGTTGTG
GbAGL6-like	Gb_41549	F	ATCCCAGATACAAACGTCACA
		R	ACAGTGCTTACAGGATGGGC
GbMADS1	Gb_36364	F	TGAAAAGTGCTCGTATGCAGTGC
		R	CCGTGTAGTTATCCTGGGGC
GbMADS8	Gb_28337	F	CGTGCATTCAGTGCTATGCAAGA
		R	GCGGAGTGGGACAGGATAAA
GbC3HDZ	Gb_18245 Gb_22761 Gb_02083	F	GGCTACMGGAACTGCTGTT
		R	GGRGGYACATTCTGTAAWAGC
GbiYAB1B	Gb_22423	F	GCCAGAGATGTCGAGCAGTT
		R	TCCATCATCAATCCCAGGCG
GbiYABC	Gb_08229	F	ATGTCTACATGTATTGAGTTCAG
		R	CTTCTGCGACGGATGTAAGT
