# Supplementary figures and images for: Identification of key regulatory genes involved in the sporophyte and gametophyte development in Ginkgo biloba ovules revealed by in situ expression analyses
Source: Am J Bot. 2022 May 19;109(6):887–98. doi: 10.1002/ajb2.1862 (PMC9322462; doi:10.1002/ajb2.1862)

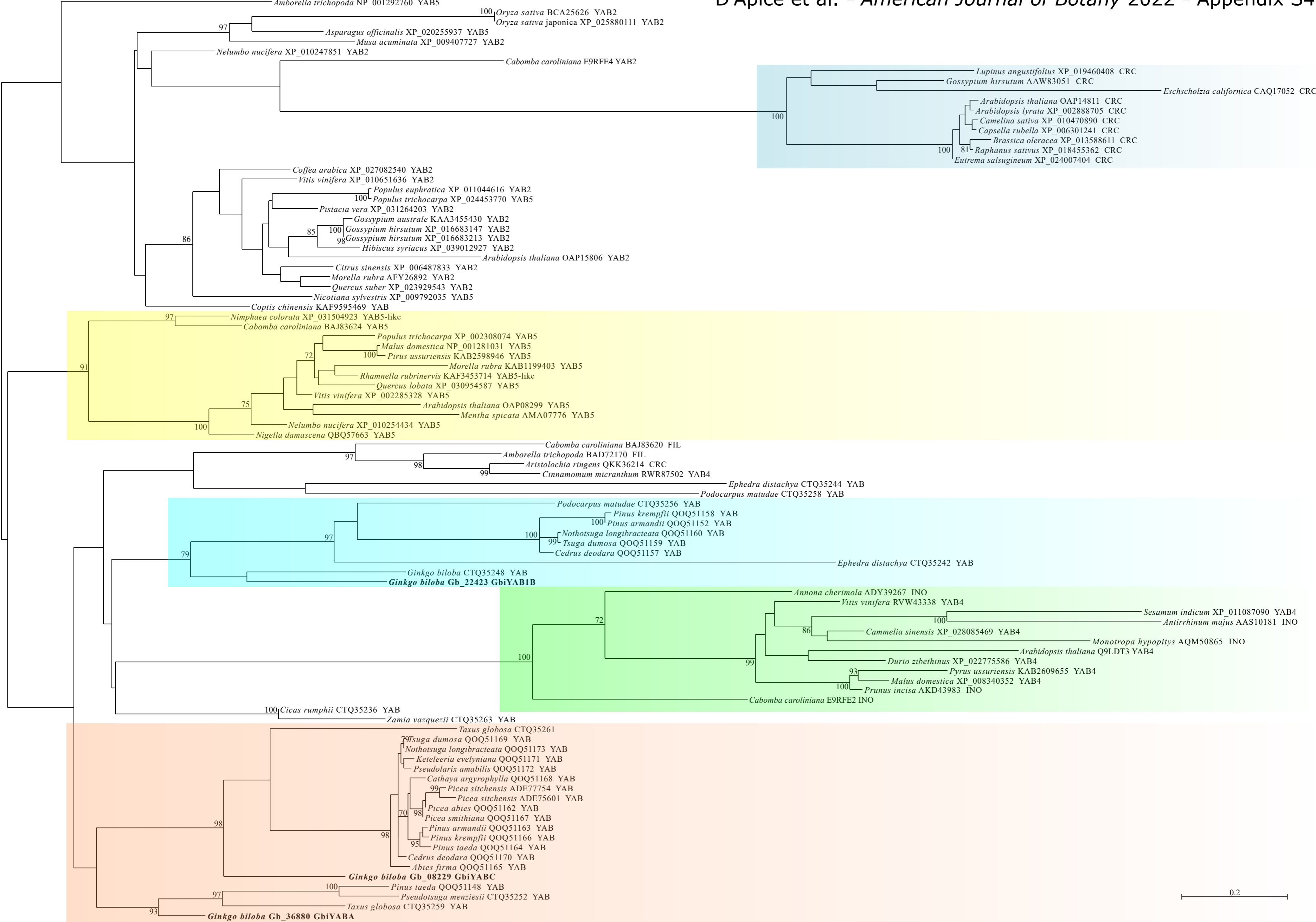

Supplement: Supplementary file 4 — Appendix S4. Maximum Likelihood (ML) phylogenetic analysis of YABBY protein sequences. 1000 bootstrap replicates, cut‐off of bootstrap values in the figure is 70%. Highlighted with colors from the top to the bottom, the CRABS CLAW (CRC) group, the YAB5 group, a well‐supported group of YABBY proteins of gymnosperms that contains GbiYAB1B, the INO group and the other group of gymnosperm YABBY sequences, which contains GbiYABC and GbiYABA. [file AJB2-109-887-s003.pdf]

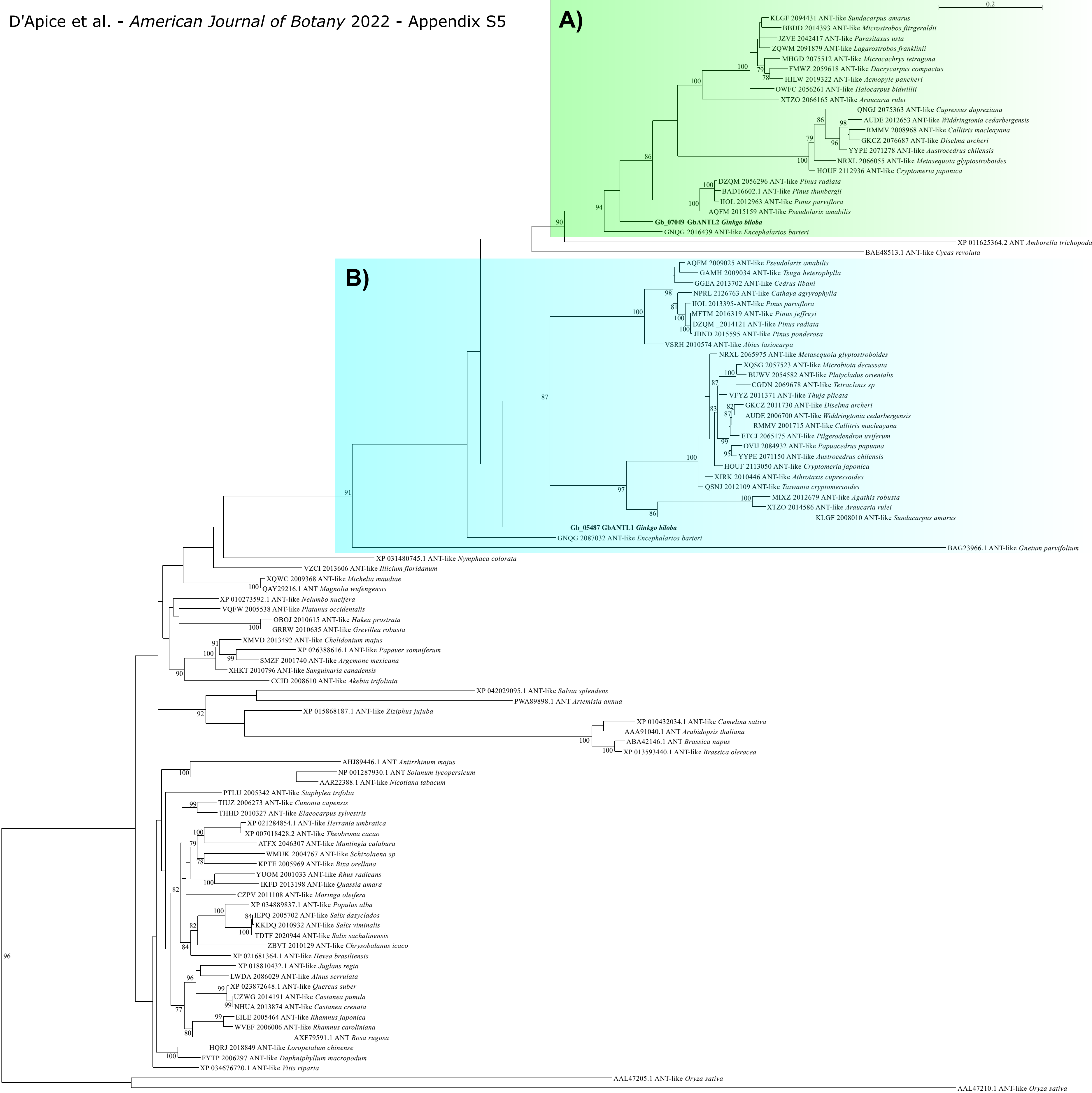

Supplement: Supplementary file 5 — Appendix S5. Maximum Likelihood (ML) phylogenetic analysis of AINTEGUMENTA protein sequences; 1000 bootstrap replicates, cut‐off of bootstrap values in the figure is 70%. Highlighted with colors are the two well‐divided groups of ANT sequences of gymnosperms. (A) The group containing GbANTL2, and (B) the group with GbANTL1. [file AJB2-109-887-s001.pdf]
